# Highly Stretchable Composite Foams via Sustainable Utilization of Waste Tire Rubbers for Temperature-Dependent Electromagnetic Wave Absorption

**DOI:** 10.3390/molecules27248971

**Published:** 2022-12-16

**Authors:** Jiajia Zheng, Mohammed Hanshe, Weiwei He, Tianyi Hang, Zhihui Li, Shaohua Jiang, Shiju E, Xiping Li, Yiming Chen

**Affiliations:** 1 Key Laboratory of Urban Rail Transit Intelligent Operation and Maintenance Technology & Equipment of Zhejiang Province, College of Engineering, Zhejiang Normal University, Jinhua 321004, China; 2 Jiangsu Co-Innovation Center of Efficient Processing and Utilization of Forest Resources, International Innovation Center for Forest Chemicals and Materials, College of Materials Science and Engineering, Nanjing Forestry University, Nanjing 210037, China

**Keywords:** porous foam, waste tire rubber, microwave absorption, mechanical property

## Abstract

Recently, the sustainable utilization of waste resources has become a low-cost and effective strategy to design high-performance functional materials to solve the increasingly serious environmental pollution problem. Herein, the flexible and highly stretchable polyurethane (PU) composite foams assisted by one-dimensional carbon nanotubes (CNTs) and zero-dimensional Fe_3_O_4_ were fabricated using waste tire rubbers (WTRs) as reinforcements during a simple self-foaming process. The collaborative introduction of conductive CNTs, magnetic Fe_3_O_4_, and WTRs with three-dimensional cross-linked structures enabled the construction of an efficient electronic transmission path and heterointerfaces inside the composite foam. The resulting composite foam possessed a desired minimum reflection loss (RL_min_) of −47.43 dB, and also exhibited superior mechanical properties with a tensile strength of >3 MPa and multiple tensile deformation recovery abilities. In addition, increasing the temperature could significantly improve the electromagnetic wave absorption performance of the composite foam. This comprehensive composite foam derived from WTRs has shown a promising development potential for using waste materials to relieve electromagnetic pollution.

## 1. Introduction

Nowadays, waste tire rubbers (WTRs) derived from the consumption of a large number of tires, also called black pollution, have hazardous impacts on the human health and environment [1,2]. Traditional solid waste treatment procedures, e.g., underground burial and burning of WTRs, will release an enormous amount of CO_2_ and other toxic substances, which may cause more serious secondary pollution [3,4]. Therefore, a trend from simple abandonment to sustainable reuse of WTRs is booming. The enhancements in characteristics and the environmental benefits of using WTRs have stimulated more research into developing green high-performance functional materials in the field of architecture [5] and energy storage [6].

With the advent of the 5G era, various electronic devices, e.g., smartphones and intelligent home appliances have ushered in rapid development, greatly facilitating people’s lives. However, the exponential growth in the power of radio frequency equipment also results in a significant increase in ground electromagnetic radiation, which brings about the increasingly serious problem of electromagnetic pollution [7,8,9]. Therefore, developing effectual electromagnetic protection materials has become a hot research topic. In view of the inherent three-dimensional (3D) cross-linked structure and high content of carbon black [10,11], the WTRs can also be considered as potential reinforcements to construct effective electromagnetic interference shielding or electromagnetic wave absorption (EWA) composite materials, by introducing various functional fillers [3,12] to alleviate the electromagnetic pollution. For example, Sheng et al. found that the WTRs could facilitate good distribution of the Ni layers at the interfaces and promote the effective establishment of the conductive network inside the material, thus offering the effective attenuation of electromagnetic waves (EWs) [10]. In our previous research [13] we fabricated an EWA composite composed of the tadpole-like carbon nanotube (CNT)/Fe_3_O_4_ and WTRs. The one-dimensional CNTs with high aspect ratio and good mechanical properties could form effective conductive networks inside the composite to attenuate EWs, owing to excellent conductivity and dielectric loss ability of CNTs [14]. While as an eco-friendly magnetic nanomaterial, zero-dimensional Fe_3_O_4_, with a feature of ease of synthesis, showed an outstanding magnetic loss property for EWs because free electrons could be exchanged rapidly between iron ions distributed in an octahedral structure [15]. Therefore, the resulting composite could exhibit an ideal EWA capability owing to the synergistic effect of the dielectric and magnetic losses. However, the mechanical instability and difficulty in self-support limited its application scopes. In order to meet realistic application requirements, a reliable route is to design comprehensive and particularly durable 3D elastomer materials (e.g., aerogels, foams) [16,17,18,19], which combines good EWA properties with excellent mechanical properties while tolerating external environmental changes.

**Figure 1:**
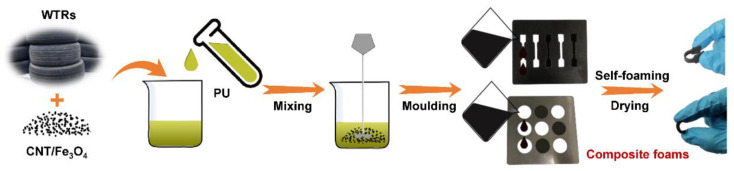
The schematic diagram of fabricating the PU/WTR@CNT/Fe_3_O_4_ composite foams.

For this reason, in this work, the polyurethane (PU) possessing excellent characteristics of low cost, corrosion resistance, and flexibility was exploited as an elastomer matrix [20,21] which could endow the WTR@CNT/Fe_3_O_4_ composites with superior mechanical strength and durability, thus developing a highly stretchable, porous, and self-foaming composite foam (Figure 1). The morphology and structure, mechanical performances, electromagnetic parameters, and EWA performances of the composite foam were investigated in detail. Furthermore, its temperature dependence on the EWA characteristics was evaluated and the possible EWA mechanism was also explained. Such a high-performance composite foam derived from the sustainable utilization of WTRs is expected to be assembled into a promising and performance-adjustable electronic device for different realistic scenarios, which can effectively mitigate electromagnetic pollution while promoting waste reutilization.

## 2. Results and Discussion

### 2.1. Morphology and Structure

**Figure 2:**
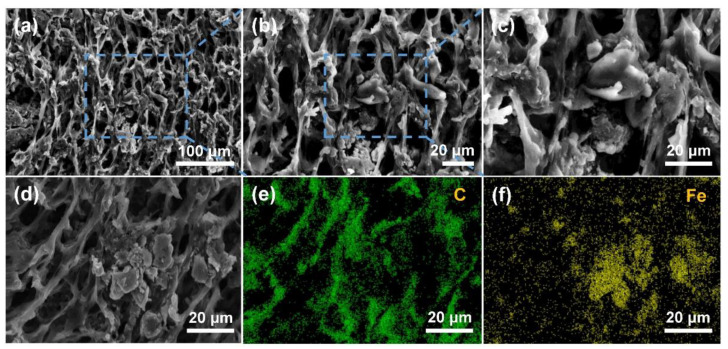
(**a**–**d**) SEM images of the PU/WTR@CNT/Fe_3_O_4_ composite foam with (**e**) C, and (**f**) Fe element mapping.

**Figure 3:**
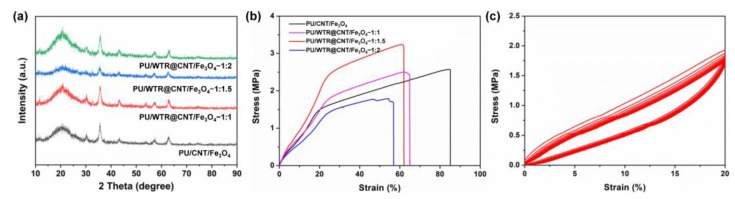
(**a**) XRD patterns and (**b**) tensile stress–strain curves of the PU/CNT/Fe_3_O_4_ and PU/WTR@CNT/Fe_3_O_4_ composite foams; (**c**) Ten stretch–release cycles of the PU/WTR@CNT/Fe_3_O_4_—1:1.5 composite foam.

The PU/WTR@CNT/Fe_3_O_4_ composite foam showed a 3D porous network structure with a pore size of <15 μm after moisture self-foaming (Figure 2a). Its rough surface came from the complex structure of WTRs (Figure 2b) [12]. The cross-linked WTRs helped to connect and bind the CNT/Fe_3_O_4_ nanoparticles into the PU matrix (Figure 2c,d). The presence of this porous structure could enhance the multiple reflections of the EWs inside the composite foam, which was highly beneficial to the energy consumption of EWs. The C (Figure 2e) and Fe (Figure 2f) elements could also be observed on the pore wall of the foam and EDS spectrum (Figure S1), implying the effective embedding of WTRs, CNTs, and Fe_3_O_4_. In addition, the XRD patterns of the PU/CNT/Fe_3_O_4_ and PU/WTR@CNT/Fe_3_O_4_ composite foams are exhibited in Figure 3a. The characteristic peaks of each foam appeared at the positions of 2θ = 30.3°, 35.6°, 43.1°, 57.3°, and 63.0°, which corresponded to (220), (311), (400), (511), and (440) planes of Fe_3_O_4_, respectively [22]. A weak peak at 2θ = 26.2° was attributed to the graphite (002) crystal plane of CNTs [23]. The above showed that the introduction of WTRs and PU basically did not affect the crystalline structure of the CNT/Fe_3_O_4_ nanoparticles.

Because the materials are exposed to external stresses during their practical use [24], it is necessary to evaluate the mechanical properties of the PU/WTR@CNT/Fe_3_O_4_ composite foams. With the increase in the WTR content, although the elongation at break of the composite foam has decreased, the tensile strength could be greatly improved and reach ~3.2 MPa for the PU/WTR@CNT/Fe_3_O_4_—1:1.5 composite foam (Figure 3b), which was better than PU/CNT/Fe_3_O_4_ by 30%. It was due to the 3D cross-linked structure similar to high-viscosity gel and the enhancement effect of the low-cost and broadly sourced WTRs inside PU matrix [10]. However, as WTRs continued to increase the mechanical property of the foam would drop, probably owing to excessive WTRs inside PU. Particularly, the PU/WTR@CNT/Fe_3_O_4_—1:1.5 composite foam could still almost return to its original state even after ten stretch–release cycles (Figure 3c), demonstrating its required stretchability and durability.

### 2.2. EWA Properties

**Figure 4:**
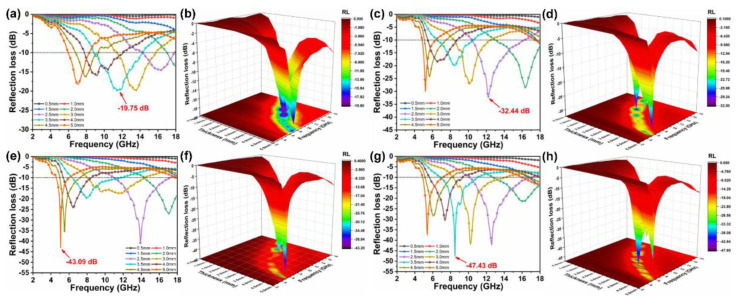
The RL of the composite foams with different thicknesses at different frequencies: (**a**,**b**) PU/CNT/Fe_3_O_4_, (**c**,**d**) PU/WTR@CNT/Fe_3_O_4_—1:1, (**e**,**f**) PU/WTR@CNT/Fe_3_O_4_—1:1.5, and (**g**,**h**) PU/WTR@CNT/Fe_3_O_4_—1:2.

The EWA properties of the PU/WTR@CNT/Fe_3_O_4_ composite foams were evaluated (Figure 4) according to reflection loss (RL) values by combining permittivity (ε_r_) with permeability (μ_r_) as the following equations [25]:

(1) $$Z_{\mathrm{in}}{=Z}_{0}\sqrt{\frac{\mu_{r}}{\varepsilon_{r}}}\tan h\left[j\frac{2\pi \mathrm{fd}}{c}\sqrt{\mu_{r}\varepsilon_{r}}\right]$$

(2) $$\mathrm{RL}\;(\mathrm{dB})=20\log\left|\frac{Z_{\mathrm{in}}{-Z}_{0}}{Z_{\mathrm{in}}{+Z}_{0}}\right|$$

where f, d, c, Z_in_, and Z_0_ are the frequency, thickness of the absorber, light velocity, input impedance on the surface of the absorber, and air impedance, respectively. The RL value of < −10 dB is required, meaning that more than 90% of the incoming EWs may be absorbed [26].

As shown in Figure 4a,b, the PU/CNT/Fe_3_O_4_ composite foam without WTRs showed a limited minimum RL (RL_min_) value of −19.75 dB at 11.6 GHz. With the introduction of WTRs and content increase, the EWA performances of the composite foam could be effectively improved and its RL_min_ peak moved to the low band. When the mass ratio of CNT/Fe_3_O_4_ and WTRs was 1:2, a superior RL_min_ value of −47.43 dB with a matching thickness of 3.5 mm at 8.5 GHz and wide effective absorption bandwidth of 4.2 GHz (6.9–11.1 GHz) was obtained from the PU/WTR@CNT/Fe_3_O_4_—1:2 composite foam (Figure 4g,h). Furthermore, the effective absorption bandwidth and absorption peak of the composite foam could be effectively controlled by adjusting the corresponding matching thickness. This ideal EWA characteristic might be attributed to the good synergism between dielectric loss and magnetic loss caused by 1D CNTs and 0D Fe_3_O_4_ assisted by WTRs to construct segregated structures inside the composite foam, which facilitated the effective attenuation and absorption of EWs.

**Figure 5:**
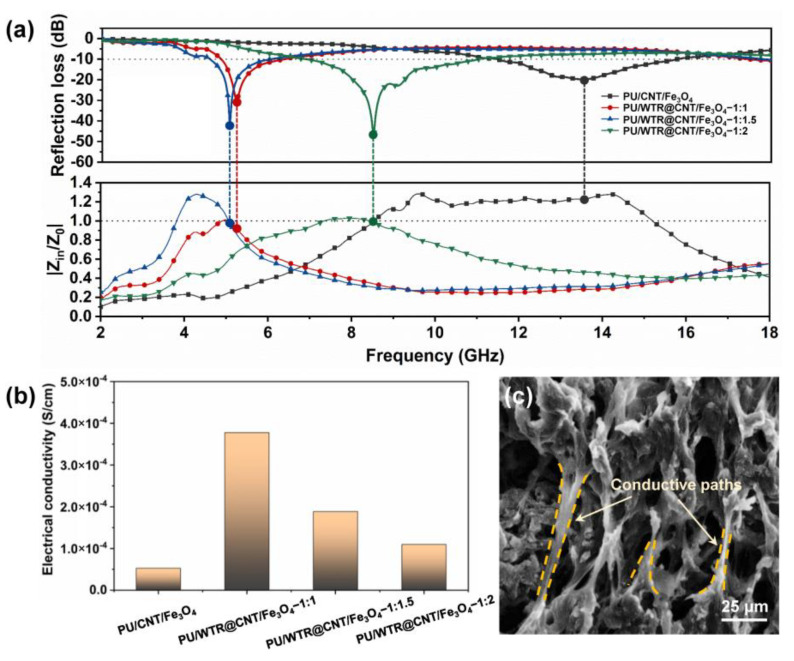
(**a**) The RL and |Z_in_/Z_0_| of the composite foams at different frequencies. (**b**) The σ and (**c**) conductive network structure of the composite foams.

A good impedance matching confirms that the EWs are more easily entering the material, leading to better EWA capacity. The |Z_in_/Z_0_| values should be near or equal to one, indicating that the vast majority of incident EWs can pass through the material without being reflected at the air-absorber surface [27]. Apparently, the impedance matching of the PU/WTR@CNT/Fe_3_O_4_—1:2 composite foam was closer to 1 compared to that of the PU/CNT/Fe_3_O_4_ (Figure 5a), indicating its more favorable absorption for EWs. Moreover, the electrical conductivity (σ) is an essential embodiment of EWA performances, as shown in Figure 5b. The addition of WTRs would greatly improve the electrical properties of the PU-based composite foams because of the presence of conductive carbon black inside WTRs [28], which could promote the establishment of more continuous and efficient conductive paths with the participation of CNT/Fe_3_O_4_ nanoparticles and PU elastic matrix (Figure 5c) [12]. Meanwhile, the continued increase in WTRs would instead reduce the σ of the composite foams owing to the aggregation of excess WTRs and the increase in non-conductive components inside WTRs. However, it in turn was more conducive to the improvement of the EWA performances. This was because for the attenuation of EWs, too high or too low σ was unfavorable, and only an appropriate σ could help to better match the interface impedance [29].

### 2.3. EWA Mechanisms

**Figure 6:**
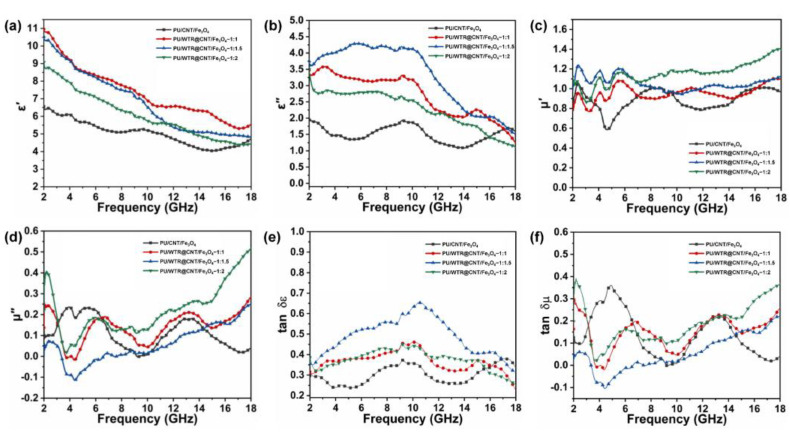
The (**a**) ε′, (**b**) ε″, (**c**) μ′, (**d**) μ″, (**e**) tan δε, and (**f**) tan δμ of the PU/CNT/Fe_3_O_4_ and PU/WTR@CNT/Fe_3_O_4_ composite foams at different frequencies.

According to Maxwell’s theory, the ε_r_ consists of the real part (ε′) and imaginary part (ε″), and the μ_r_ is made up of the real part (μ′) and imaginary part (μ″), while the fundamental ε′ and μ′ represent the storage capacity of electrical and magnetic energy, and the ε″ and μ″ are the dissipation capacity of electrical and magnetic power, respectively [30,31]. As can be seen from Figure 6a,b, an overall declining tendency with increasing frequency from 2 to 18 GHz described the complex ε altering ε′ and ε″ of different composite foams, as a result of the frequency dispersion behavior [32]. It was evident that the ε″ value of the PU/CNT/Fe_3_O_4_ composite foam without WTRs had the lowest result compared to other samples, due to the increase in the σ and dielectric loss caused by the addition of WTRs inside other foams according to the free electron theory (ε″ = σ/2ε_0_πf), which might be logically understood [33]. In addition, the ε″ curves of the composite foams fluctuated (Figure 6b), indicating the generation of the inherent resonance peaks from the dipolar polarization related to the CNT/Fe_3_O_4_ nanoparticles and interface polarization at the interfaces owing to numerous charge accumulations between fillers and matrix [34,35]. According to Debye theory, the Cole–Cole plots will demonstrate semicircular shapes if polarization losses occur based on equation S1 [36,37]. As shown in Figure S2, all composite foams exhibited several semicircles, indicating the existence of multiple Debye relaxations, which helped to improve the dielectric properties [38]. In addition, with the introduction of WTRs more semicircles and a long straight tail appeared in the PU/WTR@CNT/Fe_3_O_4_—1:2 composite foam (Figure S2d), suggesting more enhanced conduction losses [39]. For μ_r_, it was found that the μ′ and μ″ curves were unstable, showing a trend of slight decrease and then increase with increasing frequencies (Figure 6c,d). The increased contents of WTRs possessed a smaller effect on μ′ and μ″ than ε′ and ε″ of the composite foam, which was caused by magnetic losses provided by Fe_3_O_4_, including natural resonance at low frequency, eddy current loss and exchange resonance at >10 GHz [40].

Furthermore, the dielectric loss tangent (tan δε = ε″/ε′) and magnetic loss tangent (tan δμ = μ″/μ′) were used to evaluate the contribution of dielectric loss and magnetic loss for EWA of composite foams at different frequencies. Generally, the effective absorption of EWs of the composite foam was a result of the synergy between dielectric loss and magnetic loss. The value of tan δε was higher than that of tan δμ overall (Figure 6e,f), revealing the dominant position of dielectric loss for the PU/WTR@CNT/Fe_3_O_4_ composite foam. Particularly, the attenuation effect of EWs can be assessed by the attenuation constant (α) based on Equation (S2) [41]. With the increase in WTRs, the α values of the composite foams also gradually increased (Figure S3). This was attributed to the adapted electromagnetic features and a 3D porous heterogeneous structure composed of CNTs, Fe_3_O_4_, and WTRs inside the PU matrix, which facilitated the strong attenuation for EWs.

**Figure 7:**
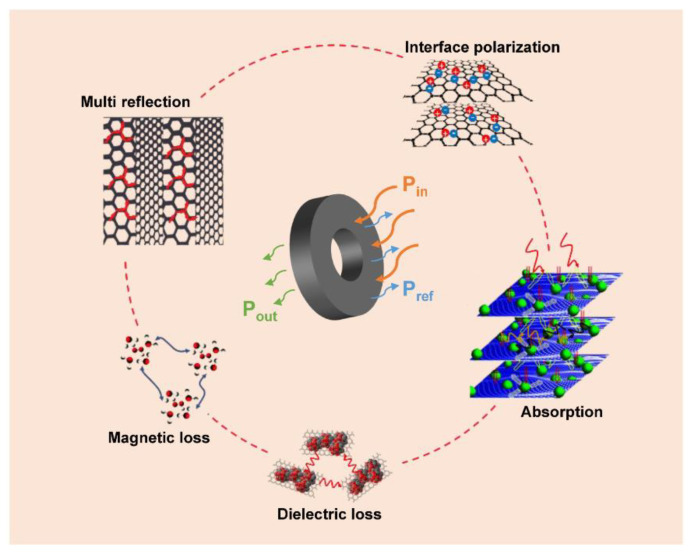
The EWA mechanisms of the PU/WTR@CNT/Fe_3_O_4_ composite foam.

To sum up, the EWA mechanisms of the PU/WTR@CNT/Fe_3_O_4_ composite foam were interpreted in Figure 7. When the EWs came into contact with the foam surface, except for a small portion that was reflected back, most of them entered the interior of the material due to the good interface impedance matching. Coupling WTRs, CNTs, and Fe_3_O_4_ embedding in the PU matrix with porous structures was responsible for the efficient attenuation of EWs inside the composite foam. Firstly, conductive carbon black in WTRs and CNTs acted as conductive paths for charge transmissions, providing enhanced conductivity and considerable dielectric losses. Secondly, the introduction of Fe_3_O_4_ nanoparticles supplied good magnetic properties and magnetic losses. In addition, a large number of heterogeneous interfaces were created among CNT/Fe_3_O_4_ nanoparticles, WTRs, and PU, increasing interface polarization and relaxation losses. Next, the interior 3D porous feature in the composite foam greatly aided in multiple reflections and scattering of EWs. Finally, the absorbed EWs were consumed by converting them into heat energy or other forms of energy.

### 2.4. Temperature Dependence of EWA

**Figure 8:**
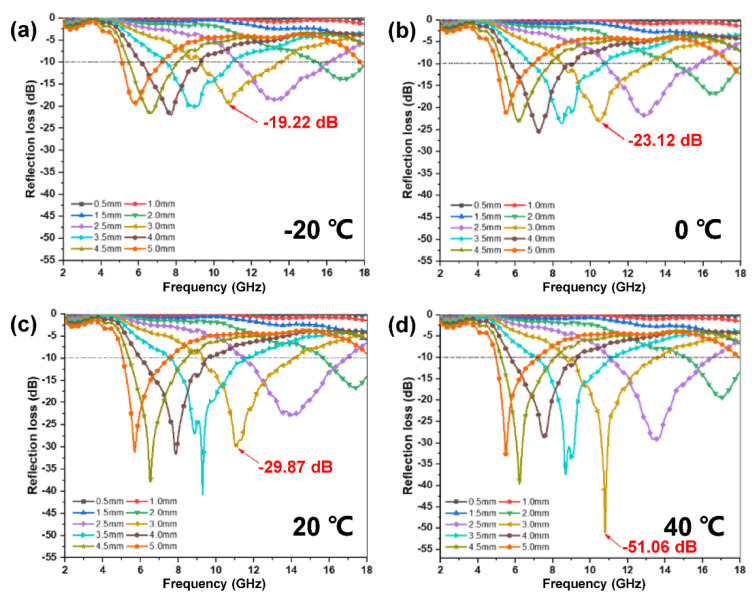
The EWA temperature dependence of the PU/WTR@CNT/Fe_3_O_4_—1:2 composite foam at (**a**) −20 °C, (**b**) 0 °C, (**c**) 20 °C, and (**d**) 40 °C.

Designing a high-performance material with tunable EWA characteristics under the stimulation of external temperature is still a significant challenge. Therefore, the EWA performances of the PU/WTR@CNT/Fe_3_O_4_—1:2 composite foam were investigated at different temperatures to evaluate the EWA temperature dependence of the material (Figure 8).

By changing the external temperature, the EWA performances of the composite foam could be regulated. When the temperature was −20 °C, a low RL_min_ reached only about −20 dB (Figure 8a). With the increase in temperature, the EWA performances of the composite foam also rose significantly, accompanied by an increase in ε′ and ε″ (Figure S4a,b). The highest RL_min_ value of −51.06 dB could be obtained at 40 °C (Figure 8d). In particular, the μ′ and μ″ values of the foam did not change significantly with adjusting temperatures (Figure S4c,d), indicating that the thermal stimulation had a weak influence on the magnetic loss. Such a characteristic was because when the external temperature rose, the air filled between the porous structures inside the composite foam would be heated and expanded. The resulting pressure could squeeze the pore wall, thus shortening the distance between conductive fillers. Then it was bound to increase the contact between WTRs and CNTs to promote the formation of a more effective conductive network for the migration of free electrons and charge accumulations generating more interface polarizations inside the PU-based composite foam, which was beneficial to the rapid decay of EWs. On the contrary, due to the decreased electron activities and evacuation of the conductive fillers under a low temperature, the attenuation ability of the composite foam to EWs was reduced, resulting in weakening its EWA performances.

## 3. Materials and Methods

### 3.1. Materials

WTRs and acidified CNTs were supplied by ENOCH GRASS Co., Ltd. (Guangdong, China) and Nanocyl SA (Sambreville, Belgium), respectively. PU was provided by Jining Huakai Resin Co., Ltd. (Jining, China). NH_4_Fe(SO_4_)_2_·12H_2_O and (NH4)_2_Fe(SO_4_)_2_·6H_2_O were purchased from Tianjin Kermel Chemical Reagent Co., Ltd. (Tianjin, China) and Sinopharm Chemical Reagent Co., Ltd. (Shanghai, China), respectively. Ammonia (NH_3_·H_2_O) and Ethanol were obtained from Yonghua Chemical Co., Ltd. (Changzhou, China). Deionized water was home-made from the laboratory.

### 3.2. Fabrication of PU/WTR@CNT/Fe_3_O_4_ Composite Foams

The tadpole-like CNT/Fe_3_O_4_ nanoparticles were synthesized according to our previous approach [13]. Briefly, CNTs were added in a mixed aqueous solution (120 mL) of (NH_4_)_2_Fe(SO_4_)_2_·6H_2_O (0.96 g) and NH_4_Fe(SO_4_)_2_·12H_2_O (2.37 g) with sonicating for 30 min (XO-1200D, Nanjing Xianou Instrument Manufacturing Co., Ltd., Nanjing, China). Then, a NH_3_·H_2_O solution (25 wt%) was dropped into the above mixture for 30 min at 50 °C for the co-precipitation of Fe_3_O_4_. The products were collected after multi-washing with ethanol and deionized water, and dried at 60 °C in a DZF-6096 drying oven (Shanghai Yiheng Scientific Instrument Co., Ltd., Shanghai, China) to obtain the CNT/Fe_3_O_4_ nanoparticles.

The CNT/Fe_3_O_4_ nanoparticles were mixed with the microwave-assisted WTRs in ethanol and were sonicated for 30 min. Next, the WTR@CNT/Fe_3_O_4_ composites were collected and dried at 60 °C. Among them, the mass ratios of CNT/Fe_3_O_4_ and WTRs were set to 1:1, 1:1.5, and 1:2. The 15 wt% of WTR@CNT/Fe_3_O_4_ and 85 wt% of PU matrix were fully blended and poured into in a mold for self-foaming by completely reacting with moisture in the air at room temperature. Finally, the above products were ambient-pressure dried at 35 °C for 5 h, thus obtaining the porous PU/WTR@CNT/Fe_3_O_4_ composite foams. The PU/CNT/Fe_3_O_4_ composite foam without WTRs was also fabricated for comparison.

### 3.3. Characterization

A Hitachi S-4800 field-emission scanning electron microscope (SEM, Tokyo, Japan) was used to observe the morphology and structure of the composite foams. The X-ray diffraction (XRD) patterns of different samples were characterized by an Ultima IV X-ray diffractometer (Rigaku, Tokyo, Japan) from 2θ = 10° to 90°. A universal testing machine (UTM4204, Jinan Jiuwang Instrument Co., Ltd., Jinan, China) was applied to measure the tensile properties of the composite foams with a 30 mm/min tensile rate. The EWA parameters of the toroidal foam samples with a 7 mm outer diameter and a 3.04 mm inner diameter were recorded by a ZVB20 vector network analyzer (Rohde & Schwarz, Munich, Germany) in a temperature range from −20 °C to 40 °C.

## 4. Conclusions

In summary, the functional PU composite foams via sustainable utilization of WTRs were designed successfully using a facile self-foaming method. The electron transport channels and more abundant heterogeneous interfaces inside the composite foam could be formed due to the presence of conductive and magnetic CNT/Fe_3_O_4_ nanocomposites with a modest proportion of WTRs. The composite foam not only showed effective absorption for EWs owing to the synergetic dielectric loss and magnetic loss, but also exhibited excellent mechanical properties for multiple stretching. More importantly, the controllable adjustment of its EWA performances could be realized by changing the external temperatures. Above all, this design strategy of fabricating the high-performance PU/WTR@CNT/Fe_3_O_4_ composite foams is expected to develop ideal absorber candidates for electromagnetic protection and waste pollution mitigation.

## Data Availability

Not applicable.

## Supplementary Materials

The following supporting information can be downloaded at: https://www.mdpi.com/article/10.3390/molecules27248971/s1, Figure S1: The EDS spectrum of the PU/WTR@CNT/Fe_3_O_4_ composite foam; Figure S2: The Cole–Cole plots of the PU/CNT/Fe_3_O_4_ and PU/WTR@CNT/Fe_3_O_4_ composite foams: (a) PU/CNT/Fe_3_O_4_, (b) PU/WTR@CNT/Fe_3_O_4_—1:1, (c) PU/WTR@CNT/Fe_3_O_4_—1:1.5, and (d) PU/WTR@CNT/Fe_3_O_4_—1:2; Figure S3: The attenuation constant (α) of the PU/CNT/Fe_3_O_4_ and PU/WTR@CNT/Fe_3_O_4_ composite foams at different frequencies; Figure S4: The (a) ε′, (b) ε″, (c) μ′, and (d) μ″ of the PU/WTR@CNT/Fe_3_O_4_—1:2 composite foam at different temperatures. Equation (S1): Cole–Cole plots; Equation (S2): attenuation constant (α). Ref [37,41] are cited in the supplementary materials.
