# Supplementary material for: Highly Stretchable Composite Foams via Sustainable Utilization of Waste Tire Rubbers for Temperature-Dependent Electromagnetic Wave Absorption
Source: Molecules. 2022 Dec 16;27(24):8971. doi: 10.3390/molecules27248971 (PMC9785358; doi:10.3390/molecules27248971)
Supplement: Supplementary file 1 [file molecules-27-08971-s001.zip › molecules-2087420-supplementary.pdf]

Supporting Information

Highly Stretchable Composite Foams via Sustainable Utilization of Waste Tire Rubbers for Temperature-Dependent Electromagnetic Wave Absorption

Jiajia Zheng [1], Mohammed Hanshe [1], Weiwei He [1], Tianyi Hang [1], Zhihui Li [1], Shaohua Jiang [2], Shiju E [1], Xiping Li [1] and Yiming Chen [1,*]

1 Key Laboratory of Urban Rail Transit Intelligent Operation and Maintenance Technology & Equipment of Zhejiang Province, College of Engineering, Zhejiang Normal University, Jinhua 321004, China; jiajia.zheng@zjnu.cn (J.Z.); nea526321@gmail.com (M.H.); hww9458@zjnu.edu.cn (W.H.); hangty@zjnu.edu.cn (T.H.); lizhihui@zjnu.edu.cn (Z.L.); eshiju@163.com (S.E); lxp2010@zjnu.cn (X.L.)

2 Jiangsu Co-Innovation Center of Efficient Processing and Utilization of Forest Resources, International Innovation Center for Forest Chemicals and Materials, College of Materials Science and Engineering, Nanjing Forestry University, Nanjing 210037, China; shaohua.jiang@njfu.edu.cn

* Correspondence: yiming.chen@zjnu.edu.cn

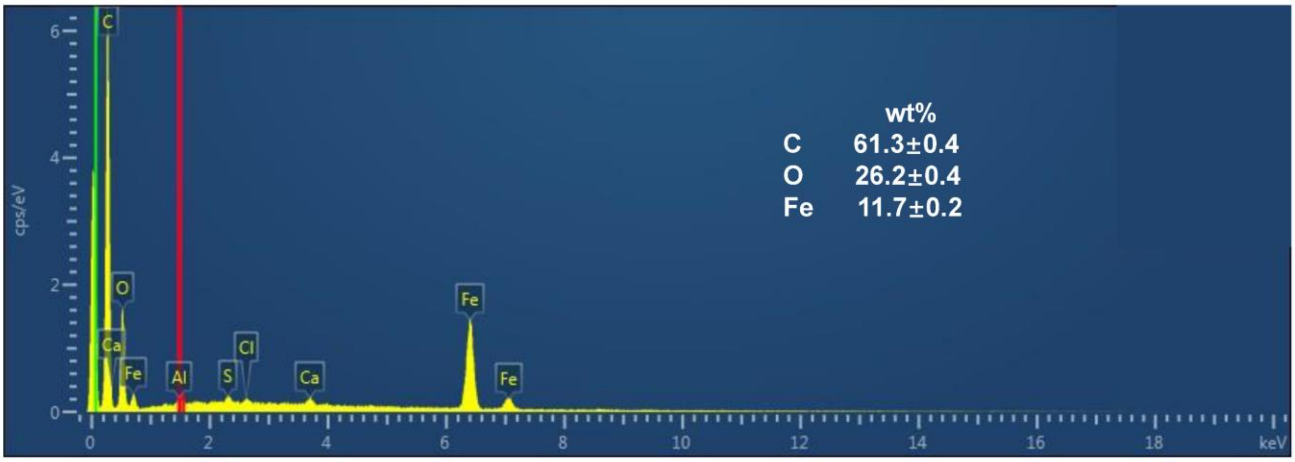

Figure S1. The EDS spectrum of the PU/WTR@CNT/Fe_3O_4 composite foam.

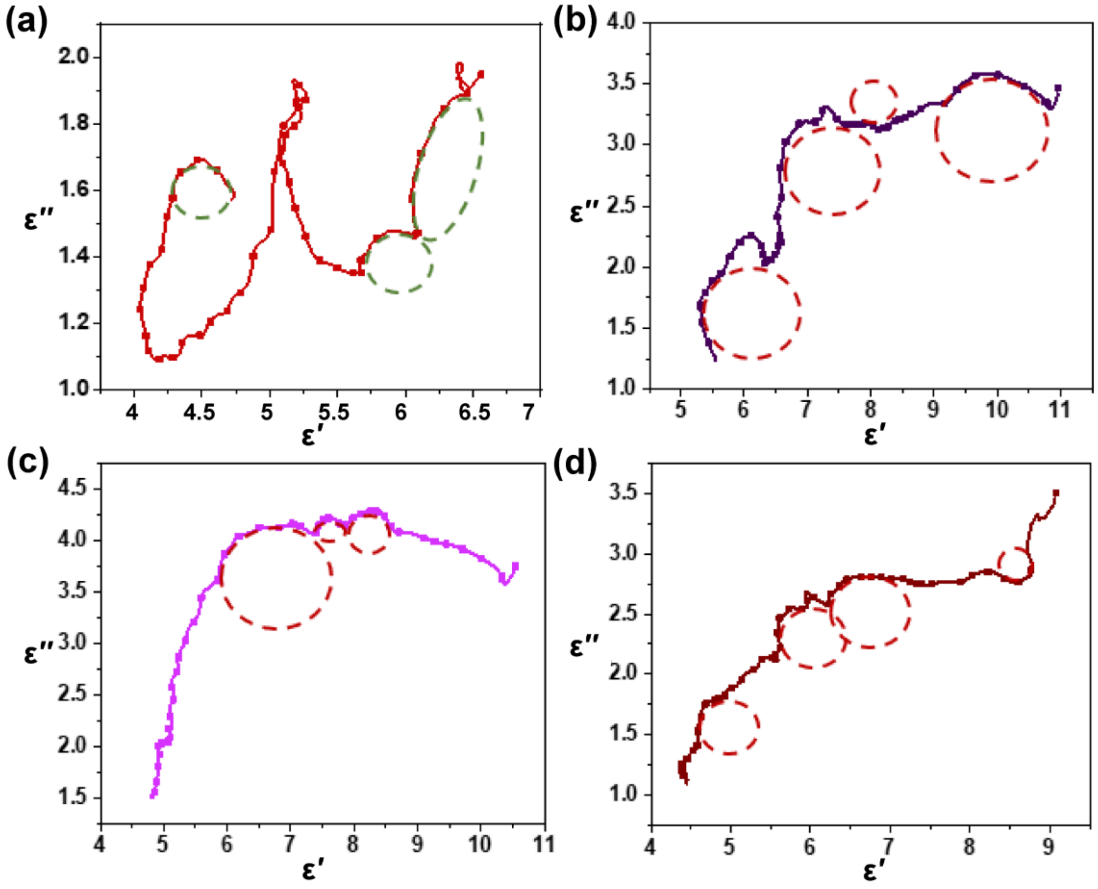

Figure S2. The Cole-Cole plots of the PU/@CNT/Fe_3O_4 and PU/WTR@CNT/Fe_3O_4 composite foams: (a) PU/@CNT/Fe_3O_4, (b) PU/WTR@CNT/Fe_3O_4-1:1, (c) PU/WTR@CNT/Fe_3O_4-1:1.5, and (d) PU/WTR@CNT/Fe_3O_4-1:2.

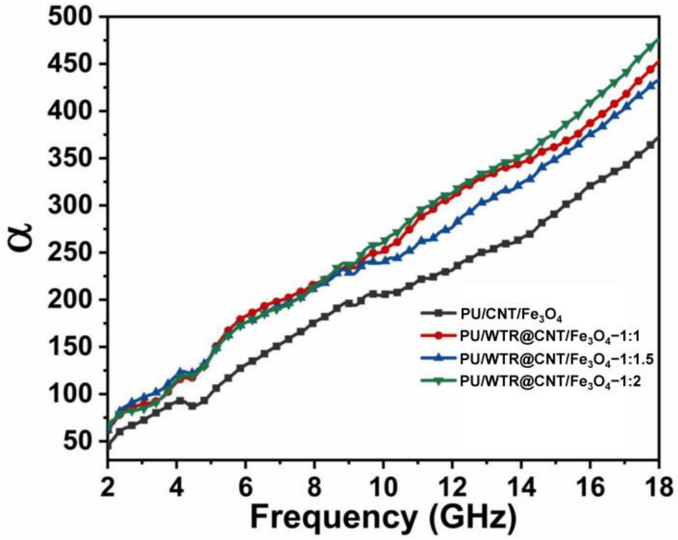

Figure S3. The attenuation constant (α) of the PU/@CNT/Fe_3O_4 and PU/WTR@CNT/Fe_3O_4 composite foams at different frequencies.

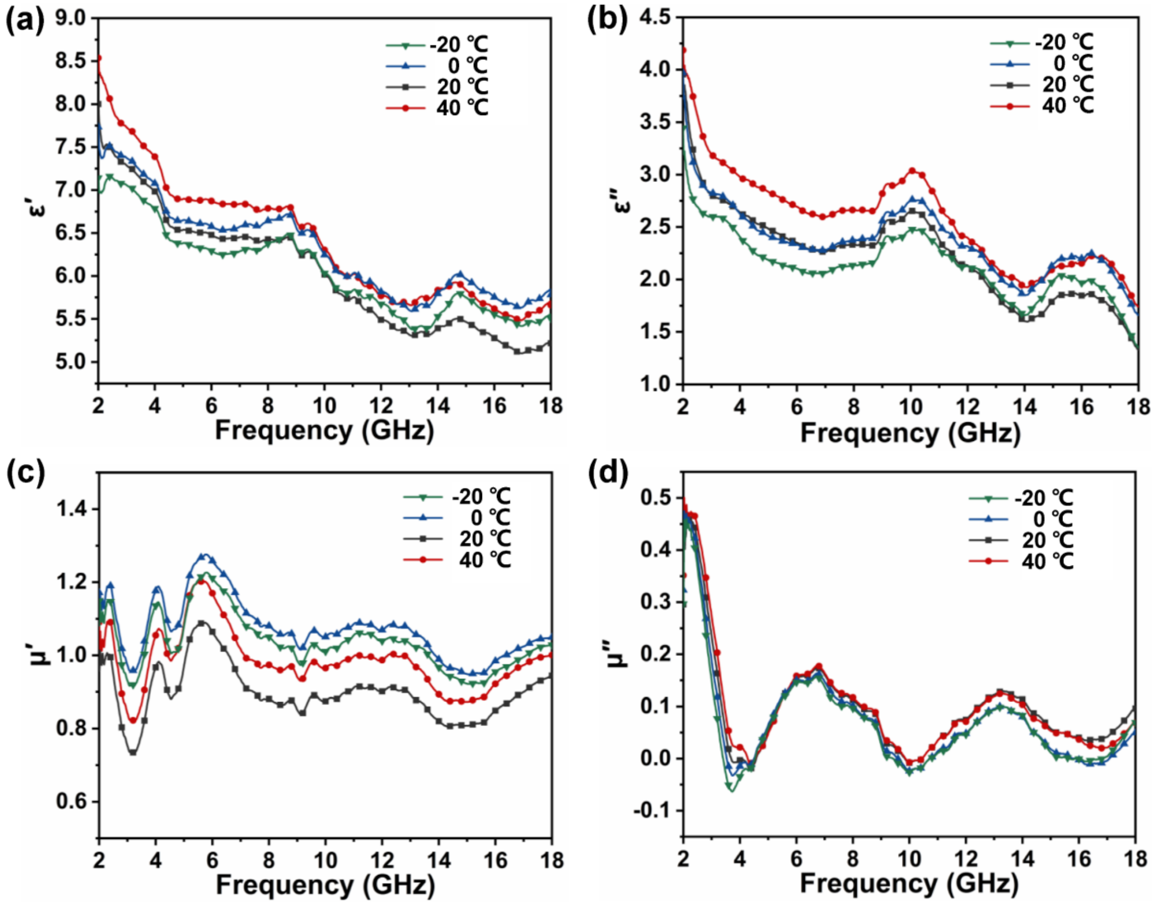

Figure S4. The (a) ε', (b) ε'', (c) μ', and (d) μ'' of the PU/WTR@CNT/Fe_3O_4-1:2 composite foam at different temperatures.

$$\left(\varepsilon' - \frac{\varepsilon_s+\varepsilon_\infty}{2}\right)^2 + (\varepsilon'')^2 = \left(\frac{\varepsilon_s-\varepsilon_\infty}{2}\right)^2 \tag{S1}$$

Where ε_s and ε_∞ are the static dielectric and relative dielectric constant at the finite frequency, respectively [1].

The attenuation constant (α) can be calculated according to the following equation [2]:

$$\alpha = \frac{\sqrt{2}\pi f}{c}\sqrt{\mu''\varepsilon''-\varepsilon'\mu'+\sqrt{(\mu''\varepsilon''-\varepsilon'\mu')^2+(\mu''\varepsilon''+\varepsilon'\mu')^2}} \tag{S2}$$

References

1. Levy, O.; Stroud, D. Maxwell Garnett theory for mixtures of anisotropic inclusions: Application to conducting polymers. *Phys. Rev. B* **1997**, *56*, (13), 8035-8046.

2. Chuai, D.; Liu, X.; Yu, R.; Ye, J.; Shi, Y. Enhanced microwave absorption properties of flake-shaped FePCB metallic glass/graphene composites. *Compos. Part A-Appl. S.* **2016**, *89*, 33-39.
